# Proteomic and phosphoproteomic profiling of SARS‐CoV‐2‐associated liver injury: a report based on rhesus macaques

**DOI:** 10.1002/mco2.358

**Published:** 2023-08-26

**Authors:** Xiaoyue Tang, Yanan Zhou, Jianqiang Wu, Peng Liu, Yehong Yang, Qiaochu Wang, Shuaiyao Lu, Jiangfeng Liu, Juntao Yang

**Affiliations:** ^1^ State Key Laboratory of Common Mechanism Research for Major Diseases Department of Biochemistry and Molecular Biology Institute of Basic Medical Sciences Chinese Academy of Medical Sciences, School of Basic Medicine Peking Union Medical College Beijing China; ^2^ State Key Laboratory of Complex Severe and Rare Diseases Peking Union Medical College Hospital Chinese Academy of Medical Sciences & Peking Union Medical College Beijing China; ^3^ National Kunming High‐level Biosafety Primate Research Center Institute of Medical Biology, Chinese Academy of Medical Sciences and Peking Union Medical College Yunnan China

Dear Editor:

Since the COVID‐19 pandemic, SARS‐CoV‐2 has continuously mutated to produce new mutant strains. The highly contagious Delta variant was originally found in December 2020,^1^ and rapidly replaced other SARS‐CoV‐2 variants, achieving global dominance by the summer of 2021. Compared with the original virus or other mutant strains, the variants B.1.1.7 (Alpha variant) and B.1.617.2 (Delta variant) are characterized by higher transmissibility and lethality, and higher prevalence rates.^2^ We would like to explore the co‐pathogenic mechanism of Delta and the original virus to find some potential protein targets for COVID‐19 treatment and also provide inspiration for the treatment of future variants.

Our study included livers from nine monkeys (three infected with the original virus [OV], three infected with Delta virus [DV], and three healthy controls [HC]). Our early data suggested that SARS‐CoV‐2 infection in rhesus macaques can induce typical characteristics of COVID‐19 after 7 days,^3^, ^4^ and the study of omics at this time point can better reflect and highlight the correlation between COVID‐19 and other systemic tissue and organ function changes. Therefore, we euthanized all animals 7 days post infection, and liver tissues were harvested for virus load measurement, H&E staining, and proteomic and phosphoproteomic analysis (Figure 1A).

**FIGURE 1:**
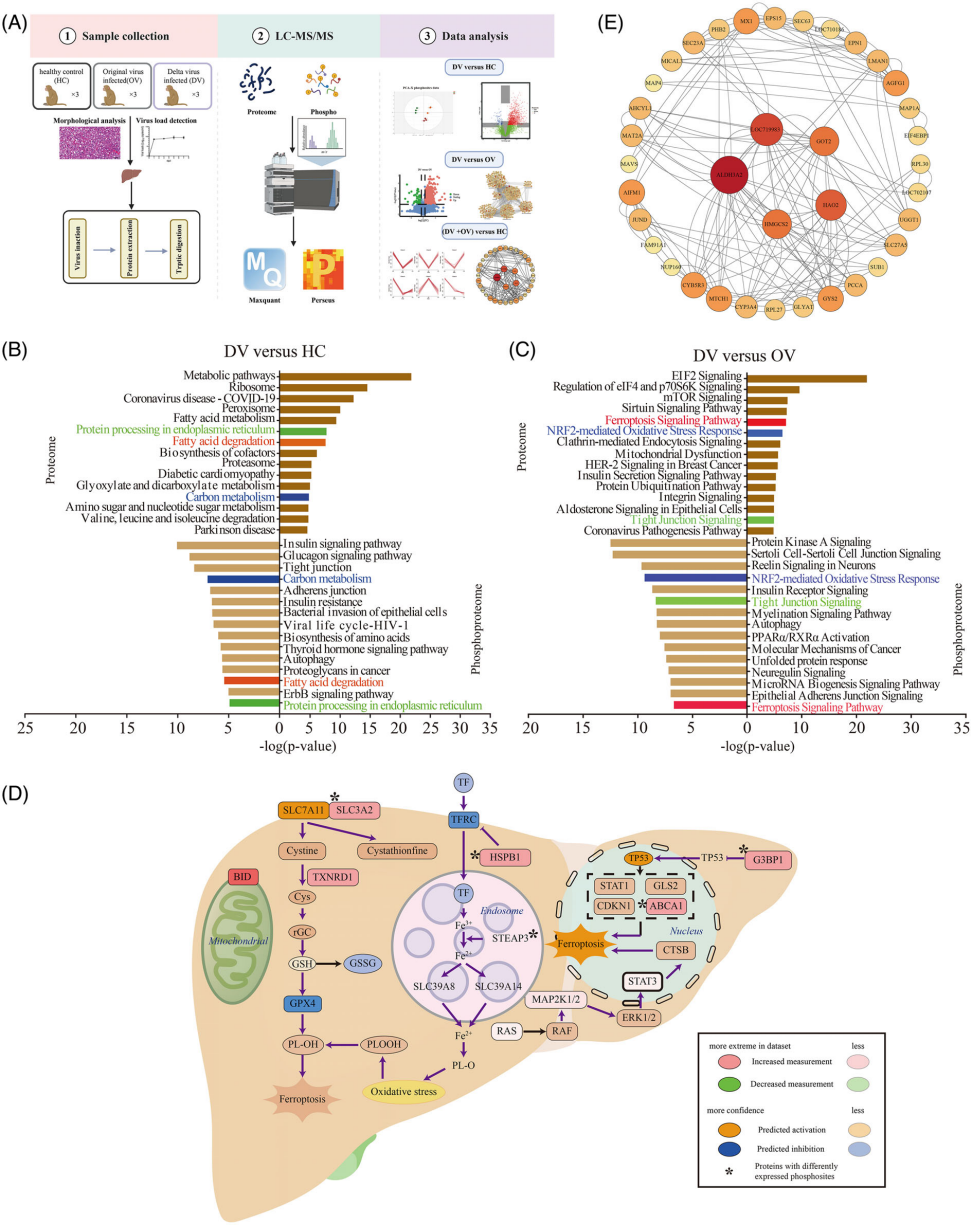
The proteomic and phosphoproteomic characterization of liver from rhesus macaques with the original COVID‐19 virus and Delta variant strain. (A) Brief workflow of this study. (B) Pathway enrichment analysis of differentially expressed proteins (top) and proteins with differently expressed phosphosites (down) in the liver of Delta virus (DV) versus healthy controls (HC) group. (C) Pathway enrichment analysis of differentially expressed proteins (top) and proteins with differently expressed phosphosites (down) in the liver of the DV versus original virus (OV) group. (D) Ferroptosis signaling pathway in livers. Red boxes: upregulated proteins in the DV groups. Green boxes: downregulated proteins in the DV groups. Orange boxes: proteins predicted to be activated in the DV groups. Blue boxes: proteins predicted to be inhabited in the DV groups. Asterisk: proteins with differently expressed phosphosites. (E) Protein–protein interaction (PPI) analysis among 45 proteins that were differently expressed in DV and OV groups when compared with HC, respectively. They also had differently expressed phosphosites in DV and OV groups when compared with HC, respectively.

SARS‐CoV‐2 RNA was detected in liver homogenate, and the mean viral load of OV and DV groups was 6.6 × 10^4^ and 7.0 × 10^4^ copies/g, respectively. H&E staining showed hepatocyte edema, hepatic hemorrhage, and scattered infiltration of inflammatory cells in the infected livers. The pathological lesion of the liver after infection of Delta was more severe than that of the original virus strain (Figure S1). Therefore, all the above information showed liver injury in the animals used in our study.

First, DV and HC groups were compared to explore liver protein and phosphosite level changes after Delta infection. For the proteomic analysis, 7540 and 7332 proteins in the DV and HC groups were quantified, respectively (Figure S2A,B and Table S1). The principal components analysis (PCA) score plot (Figure S3A) suggested an apparent difference between the two groups and 1417 differentially expressed proteins DEPs) (Figure S3B and Table S2). Gene Ontology (GO) terms were mainly enriched in peptide metabolic process, translation, ribosome, and so forth, which were related to protein synthesis and metabolism (Figure S3C–E). Pathway enrichment associated with coronavirus disease‐19, protein synthesis (such as ribosome, protein in export), and metabolism were enriched (Figure 1B and Table S3). Meanwhile, we quantified 8095 phosphosites in 3170 proteins and 10,560 sites in 3668 proteins in the DV and HC groups, respectively (Figure S2C,D and Table S4). The PCA score plot (Figure S4A) suggested an apparent difference, and 6964 sites in 3080 proteins (Figure S4B and Table S5) were differentially expressed. The GO terms of these proteins with differently expressed phosphosites were enriched in actin cytoskeleton organization, actin binding, and so forth (Figure S4C). Kinase prediction analysis showed that there were 20 kinases with a *p*‐value less than 0.05, of which CLK1, CLK2, and TLK2 were activated, and others, including AKT1 and MARK4, were inhibited according to the NetworkKIN analysis (Figure S5A and Table S6). Pathway enrichment analysis showed insulin signaling pathway, tight junction, and so forth were enriched (Figure 1B). Both proteomics and phosphoproteomics data showed enrichment in fatty acid degradation, carbon metabolism, and protein processing in endoplasmic reticulum. The proteins enriched in the three pathways for proteomic data were submitted to STRING for protein–protein interaction analysis, and 25% of proteins in the network had differently expressed phosphosites (Figure S5B). Separately, proteomics data showed the enrichment of protein synthesis‐related pathways, such as ribosome and phosphorylation data showed that liver intercellular connection, such as tight junction, and adherens junction were changed after infection. The above results indicated that liver protein synthesis and metabolism were significantly activated accompanied by Delta infection, and liver intercellular connection, such as cytoskeleton and cell adhesion, were also changed (Figure 1B and Table S3).

Then we explored differences in liver proteomics and phosphoproteomics between DV and OV groups. Regarding the proteomic analysis, similarly, the PCA plot of the two groups showed a significant separation (Figure S3A), and 1220 DEPs were identified (Figure S6A and Table S2). The ingenuity pathway analysis (IPA) showed that pathways associated with protein synthesis (such as EIF2 signaling, mTOR signaling, regulation of EIF4, and p70S6K signaling) and metabolic pathways (such as mitochondrial dysfunction and the protein ubiquitination pathway) were enriched (Figure 1C and Table S3). Moreover, the ferroptosis signaling pathway was activated; GPX4 was predicted to be inhibited, and TP53 was predicted to be activated. Delta virus infection might inhibit the expression of GPX4 and activate the expression of TP53, promoting ferroptosis in liver cells (Figure 1D). The expression levels of the proteins involved in ferroptosis in our dataset were higher than those in the HC and OV group (Figure S6B). Moreover, the integrated network analysis revealed that the upregulated expressed proteins in the DV group associated with the processes of responses to the virus, including viral infection and viral entry, may cause changes in liver function, such as lipid acid and drug metabolism (Figure S6C). For the phosphoproteomic analysis, 6334 phosphosites in 2044 proteins were differently expressed between DV and OV groups (Figure S7A and Table S5). Kinase prediction analysis showed that 30 kinases had a *p*‐value less than 0.05 (Figure S7B and Table S6), and were associated with processes including necrosis and apoptosis (Figure S7C). IPA analysis showed protein kinase A signaling, insulin receptor signaling, and so forth were enriched (Figure 1C). Ferroptosis signaling pathway, NRF2‐mediated oxidative stress response, and tight junction signaling were enriched both in proteomic data and phosphoproteomic data. In ferroptosis signaling pathway, five DEPs (STEAP3, SLC3A2, etc.) also had differentially expressed phosphosites (Figure 1D). However, protein synthesis‐associated pathways were enriched with proteomics data, while autophagy, protein kinase A, and so forth were enriched with phosphoproteomics data. The above results indicated that the liver infected with the two strains showed differences in protein synthesis, metabolism, autophagy, ferroptosis, and so forth.

Finally, we compared the DV and OV groups with the HC group, respectively, to explore common changes between the two strains. The proteomic analysis showed 425 DEPs after viral infection (Figure S8A and Table S7), among which 408 proteins showed the same trend by MFUZZ analysis (Figure S8B). Similarly, the phosphosites of 1732 proteins were changed after viral infection. The Venn diagram analysis revealed 45 proteins in common (Figure S8A). The co‐expression network showed that the top proteins according to the degree score, such as ALDH3A2, GOT2, and HMGCS2, were all related to liver function (Figure 1E and Figure S8B). Therefore, the expression level of 45 proteins, including GOT2 and ALDH3A2, were changed after viral infection, reflecting the common changes in the liver after infection of the two strains of COVID‐19.

We conducted the proteomic and phosphoproteomic profiling of SARS‐CoV‐2‐associated liver injury, and our findings may represent shared characteristics of COVID‐19 infection and were not limited to Delta variant infection. The hepatobiliary system might be influenced by COVID‐19 infection; patients with chronic liver disease have been frequently affected, experiencing high morbidity and mortality. SARS‐CoV‐2 can promote existing chronic liver diseases to liver failure and activate the autoimmune liver disease.^5^ By analyzing liver proteomics after the Delta variant infection, a comprehension of the associated biological processes and pathological mechanisms that may transpire during the illness of other variant strains can be attained, and potential therapeutic targets can be revealed.

## AUTHOR CONTRIBUTIONS

Juntao Yang, Shuaiyao Lu, and Jiangfeng Liu designed the experiments. Yanan Zhou performed the operation of viral infection in animal models and detected the viral load. Qiaochu Wang performed histopathology analyses. Xiaoyue Tang, Jianqiang Wu, Peng Liu, and Yehong Yang analyzed the proteomics and phosphoproteomics data. Xiaoyue Tang wrote the letter with the help from all the other co‐authors. All authors have read and approved the final version of the manuscript.

## CONFLICT OF INTEREST STATEMENT

The authors declare they have no conflicts of interest.

## FUNDING INFORMATION

This study was supported by grants from the Chinese Academy of Medical Sciences Innovation Fund for Medical Sciences, China (CIFMS2022‐I2M‐JB‐003, CIFMS2022‐I2M‐1‐011).

## ETHICS STATEMENT

All animal procedures in this study were approved by the Institutional Animal Care and Use Committee of the Institute of Medical Biology, Chinese Academy of Medical Science (ethics number: DWSP202002 001). All animal experiments were performed in accordance with the guidelines for the National Care and Use of Animals approved by the National Animal Research Authority and the ABSL‐4 facility of the National Kunming High‐level Biosafety Primate Research Center, Yunnan, China.

## Supporting information

Supporting Information

Supporting Information

Supporting Information

Supporting Information

Supporting Information

Supporting Information

Supporting Information

Supporting Information

## Data Availability

The mass spectrometry data have been deposited to the ProteomeXchange Consortium with the dataset identifiers IPX0003167001 and IPX0003167002.
