# Supplementary material for: Proteomic and phosphoproteomic profiling of SARS‐CoV‐2‐associated liver injury: a report based on rhesus macaques
Source: MedComm (2020). 2023 Aug 26;4(5):e358. doi: 10.1002/mco2.358 (PMC10458660; doi:10.1002/mco2.358)
Supplement: Supplementary file 1 — Supporting Information [file MCO2-4-e358-s007.docx]

Proteomic and phosphoproteomic profiling of SARS-CoV-2-associated liver injury: a report based on rhesus macaques

Xiaoyue Tang^1,2#^, Yanan Zhou^3#^, Jianqiang Wu^2#^, Peng Liu^2#^, Yehong Yang^1^, Qiaochu Wang^1^, Shuaiyao Lu^3*^, Jiangfeng Liu^1*^, Juntao Yang^1*^

Correspondence to: Juntao Yang [(yangjt@pumc.edu.cn)](mailto:(yangjt@pumc.edu.cn))

Jiangfeng Liu (ljf@ibms.pumc.edu.cn)

Shuaiyao Lu (lushuaiyao-km@163.com.)

Xiaoyue Tang, Yanan Zhou, Jianqiang Wu, and Peng Liu contributed equally to this work.

1 State Key Laboratory of Common Mechanism Research for Major Diseases, Department of Biochemistry and Molecular Biology, Institute of Basic Medical Sciences Chinese Academy of Medical Sciences, School of Basic Medicine Peking Union Medical College, Beijing, 100005 China

2 State Key Laboratory of Complex Severe and Rare Disease, Peking Union Medical College Hospital, Chinese Academy of Medical Sciences & Peking Union Medical College, Beijing 100730, China.

3 National Kunming High-level Biosafety Primate Research Center, Institute of Medical Biology, Chinese Academy of Medical Sciences and Peking Union Medical College, Yunnan, China.

**This PDF file includes:**

Supplementary Materials and Methods

Supplementary Figures S1 to S7

**Other Supplementary Materials for this manuscript include the following:**

Supplementary Tables S1 to S7

**Materials and methods**

Ethics and biosafety statement

All animal procedures in this study were approved by the Institutional Animal Care and Use Committee of the Institute of Medical Biology, Chinese Academy of Medical Science (ethics number: DWSP202002 001). All animal experiments were performed in accordance with the guidelines for the National Care and Use of Animals approved by the National Animal Research Authority and the ABSL-4 facility of the National Kunming High-level Biosafety Primate Research Center, Yunnan, China.

Virus amplification and identification

SARS-CoV-2 was obtained from the Center of Disease Control and Prevention of Guangdong Province, and the original virus was a strain named “GD108#”. Viruses were amplified in Vero E6 cells, purified, and concentrated with an ultrafilter system with a 300-kDa module (Millipore, US). SARS-CoV-2 was confirmed via reverse-transcription polymerase chain reaction (RT‒PCR), sequencing, transmission electronic microscopy, and titration via a plaque assay (10^7^ plaque-forming units [PFU]/mL).

Animal experimental procedures

Nine rhesus macaques (Macaca mulatta) were used in this study, including three original virus-infected rhesus macaques (three were male, 4-7 kg, 4-5 years old), three Delta virus-infected rhesus macaques (one was male, two were female, 4-6 kg, 3-4 years old) and three healthy controls (three were male, 4-10 kg, 5-9 years old). They were divided into three groups: the virus infection group intranasally inoculated with original SARS-CoV-2 (monkey ID, HHH-1/2/3), the virus infection group intranasally inoculated with Delta SARS-CoV-2 (monkey ID, HHH-9/10/11), and the blank untreated control group (monkey ID, HHH-6/7/8). Before viral inoculation, the animals were anesthetized with ketamine (6 mg/kg). Each animal in the virus infection group was challenged with 1 mL of 5.5 × 10^5^ PFU SARS-CoV-2 (500 μL/each nostril). Animals were dissected at 7 dpi, and tissue samples were harvested for viral load, histopathology, and proteomic and phosphoproteomic analyses.

Morphological analysis

Liver tissue samples were harvested and fixed in 10% neutral-buffered formalin for 3-7 days. Formalin-fixed paraffin-embedded tissues were cut into 5 μm sections for H&E staining and histopathologic analysis.

Viral RNA extraction and quantification of the viral RNA genome

A TRIzol suspension of 400 μL swab or 100 mg tissue samples from each animal was used for RNA extraction using a Direct-zol RNA Miniprep Extraction Kit (Zymo Research, catalog No. R2052) according to the manufacturer’s instructions. Swab samples soaked in TRIzol solution were vortexed, and then the swabs were removed. The TRIzol suspension obtained from the swabs (400 μL) was used for RNA extraction, washed with 50 μL DNase/RNase-free water to elute RNA, and stored at -80 ℃. For tissue homogenization, 1 mL TRIzol was added to 100 mg tissue. The supernatant (400 μL) was centrifuged to extract the RNA template, which was washed with 50 μL DNase/RNase-free water to elute the RNA and stored at -80 ℃. Real-time PCR (RT-PCR) was used to quantify the viral genome using TaqMan Fast Virus 1-Step Master Mix (Thermo Fisher Scientific), and SARS-CoV-2 RNA was used for the standard curve. RT‒PCR was performed on a CFX384 Touch Real-Time PCR Detection System (Bio-Rad). Primers and probe sequences were derived from the N gene (forward: 5’-GGGGAACTTCTCCTGCTAGAAT-3’, reverse: 5’-CAGACATTTTGCTCTCAAGCTG-3’, probe: 5’-FAMTTGCTGCTGCTTGACAGAT-TAMRA-3’) according to the sequence recommended by the WHO and China CDC. RT‒PCR was conducted under the following conditions: 25 ℃ for 120 s, 50 ℃ for 15 min, 95 ℃ for 120 s, and 40 cycles at 95 ℃ for 5 s and 60 ℃ for 30 s.

Sample preparation and LC‒MS/MS analysis

Approximately 150 mg of tissue from each animal was used for protein extraction. The tissue samples were added to 4 volumes of lysis buffer (1% SDS, 1% protease inhibitor, and 1% phosphatase inhibitor), homogenized, and heated for virus inactivation. After ultrasonic lysis, the remaining debris was removed by centrifugation at 12,000 × *g* at 4 ℃ for 10 min. The supernatant was collected, and the protein concentration was determined with a BCA kit.

For digestion, the protein solution was reduced with 5 mM dithiothreitol for 30 min at 56 ℃ and alkylated with 11 mM iodoacetamide for 15 min at room temperature in the dark. Then, the urea concentration of the sample was diluted to less than 2 M. Trypsin was then added at a 1:50 trypsin-to-protein mass ratio for the first digestion overnight and a 1:100 trypsin-to-protein mass ratio for a second 4 h digestion. Finally, the peptides were desalted and pending subsequent peptide fractionation.

The peptide samples for proteome analysis were fractionated by high pH reverse-phase HPLC using an Agilent 300 Extend C18 column (5 μm particles, 4.6 mm ID, 250 mm length). Briefly, peptides were first separated into 60 fractions with a gradient of 8% to 32% acetonitrile in 10 mM ammonium bicarbonate pH 9 over 60 min. Then, the peptides were combined into 10 fractions and dried by vacuum centrifugation.

For phosphopeptide enrichment, peptide mixtures were first incubated with Fe-IMAC microsphere suspensions with vibration in loading buffer (50% acetonitrile/6% trifluoroacetic acid). The Fe-IMAC microspheres with enriched phosphopeptides were collected by centrifugation. To remove nonspecifically absorbed peptides, the Fe-IMAC microspheres were washed with 50% acetonitrile/6% trifluoroacetic acid and 30% acetonitrile/0.1% trifluoroacetic acid sequentially. To elute the enriched phosphopeptides, elution buffer containing 10% NH_4_OH was added, and the enriched phosphopeptides were eluted with vibration. The supernatant containing phosphopeptides was collected and lyophilized for LC‒MS/MS analysis.

An Orbitrap Exploris^TM^ 480 mass spectrometer (Thermo Fisher Scientific) equipped with an EASY-nLC 1200 UPLC system was used for tandem mass spectrometry (MS/MS) analysis. A binary buffer system consisting of buffer A (0.1% formic acid in water) and buffer B (0.1% formic acid in 90% acetonitrile) was used for peptide separation. The FAIMS device was placed between the nanoelectrospray source and the mass spectrometer. The electrospray voltage was applied at 2.3 kV. The intact peptides were detected in the orbitrap at a resolution of 60,000. Peptides were then selected for MS/MS with NCE 27, and the fragments were detected in the orbitrap at a resolution of 30,000. A data-dependent procedure alternated between one MS scan followed by 25 MS/MS scans with 20 s dynamic exclusion. For phosphoproteome analysis, the automatic gain control (AGC) was set at 100%, with an intensity threshold of 2E4 and a maximum injection time of 50 ms. For proteome analysis, the intensity threshold was set as 5E4.

Database search and bioinformatics analysis

The resulting MS/MS raw files were processed using the MaxQuant search engine (v1.6.5.0) against the Macaca mulatta database (UniProt, 20201216 released) concatenated with the reverse decoy database. Trypsin/P was specified as the cleavage enzyme allowing up to 2 missing cleavages. Carbamidomethyl (C) was specified as a fixed modification. Oxidation (M), acetylation (protein N-terminal), deamidation (NQ), and phosphorylation (STY) were specified as variable modifications. The FDR was adjusted to < 1%, and the minimum score for modified peptides was set to > 40. The minimum peptide length was set at 7. For the quantification method, a match between runs was enabled. All the other parameters in MaxQuant were set to default values.

MaxQuant-derived data were submitted to Perseus for subsequent processing. First, proteins matched to potential contaminants and reverse databases were excluded. Then, only phosphosites with localization probabilities > 0.75 were retained. Proteins/phosphosites present in > 50% of samples in the control or virus-infected group were considered quantifiable. Normalization and imputation were performed using the “Divide” (by median of column) and “Replace missing values from normal distribution” functions in Perseus. The obtained matrix was used for the differential expression analyses.

Student’s *t* test was used to determine the differentially expressed proteins/phosphosites, and the cutoff was set at *p* < 0.05. Fold change (FC) was calculated by dividing the mean values of the virus-infected group by that of the control group (FC = mean of virus-infected group/mean of control group). Proteins/phosphosites with *p* < 0.05 and FC > 1.5 were considered upregulated, and those with *p* < 0.05 and FC < 0.667 were considered downregulated.

Pattern recognition analysis, including principal component analysis (PCA), was performed using SIMCA 14.0 software (Umetrics, Sweden). Data analysis and visualization were performed using GraphPad Prism version 5.0 (Intuitive Software for Science, GraphPad Software Company, San Diego, CA, USA). Gene Ontology (GO) enrichment analyses were performed using the clusterProfiler package in R (4.1.1). SwissProt accession numbers were uploaded to IPA software (QIAGEN, USA) and KEGG for pathway enrichment analysis. Kinase prediction was performed using NetworKIN following the algorithm’s instructions based on differentially expressed phosphosites in the liver.

**Supplementary Figures**

**
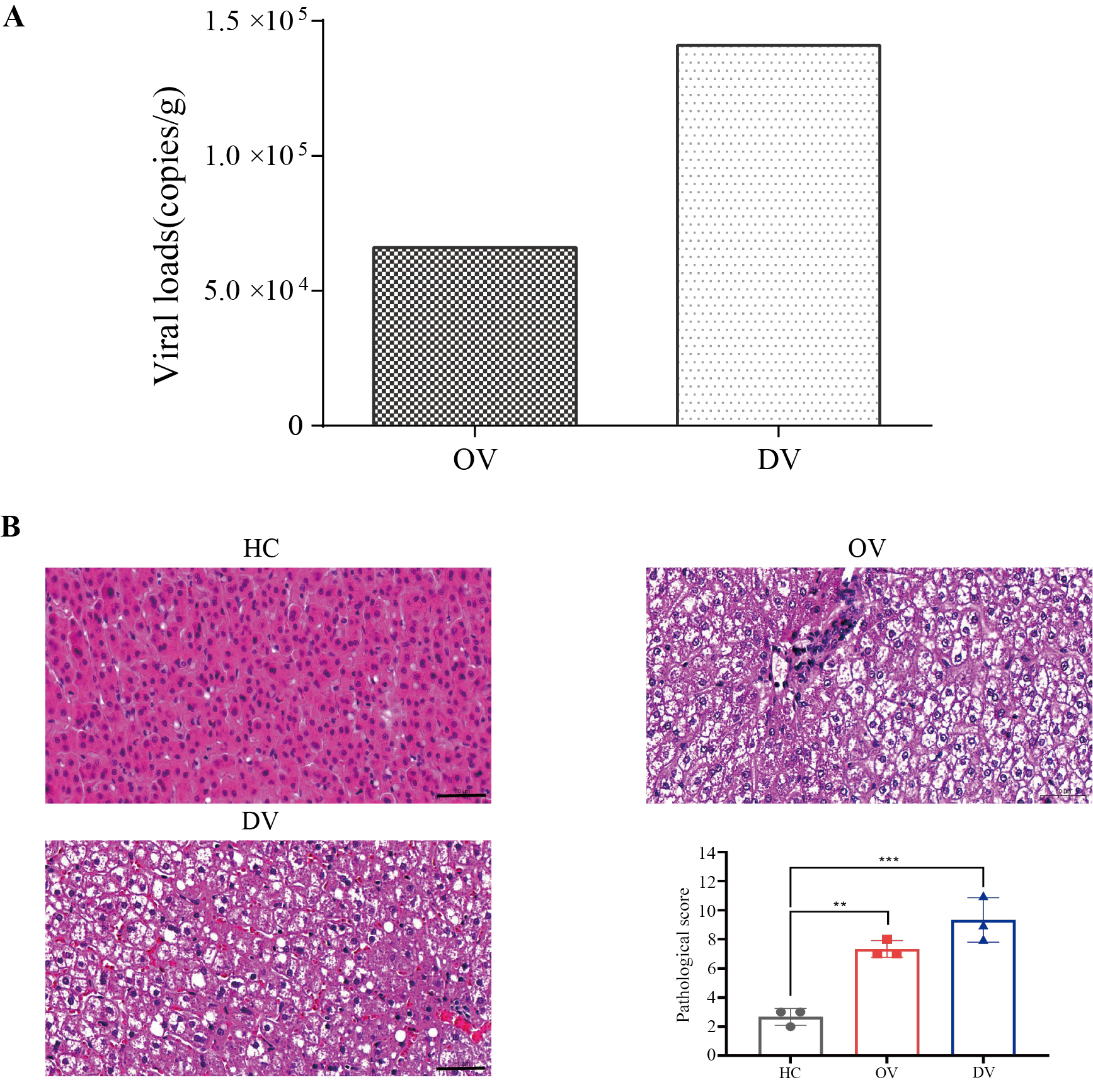
**

**Figure S1 (A)** Representative H & E staining images for healthy control (HC), original virus- infected (OV), and Delta virus-infected liver tissues in rhesus macaques (scale bar = 50 μm). In the infected livers, we observed hepatocyte edema, hepatic hemorrhage, and scattered infiltration of inflammatory cells. **(B)** The pathological score of the liver in HC, OV, and DV groups. The pathological lesion of the liver after infection of Delta was more serious than that of the original virus.


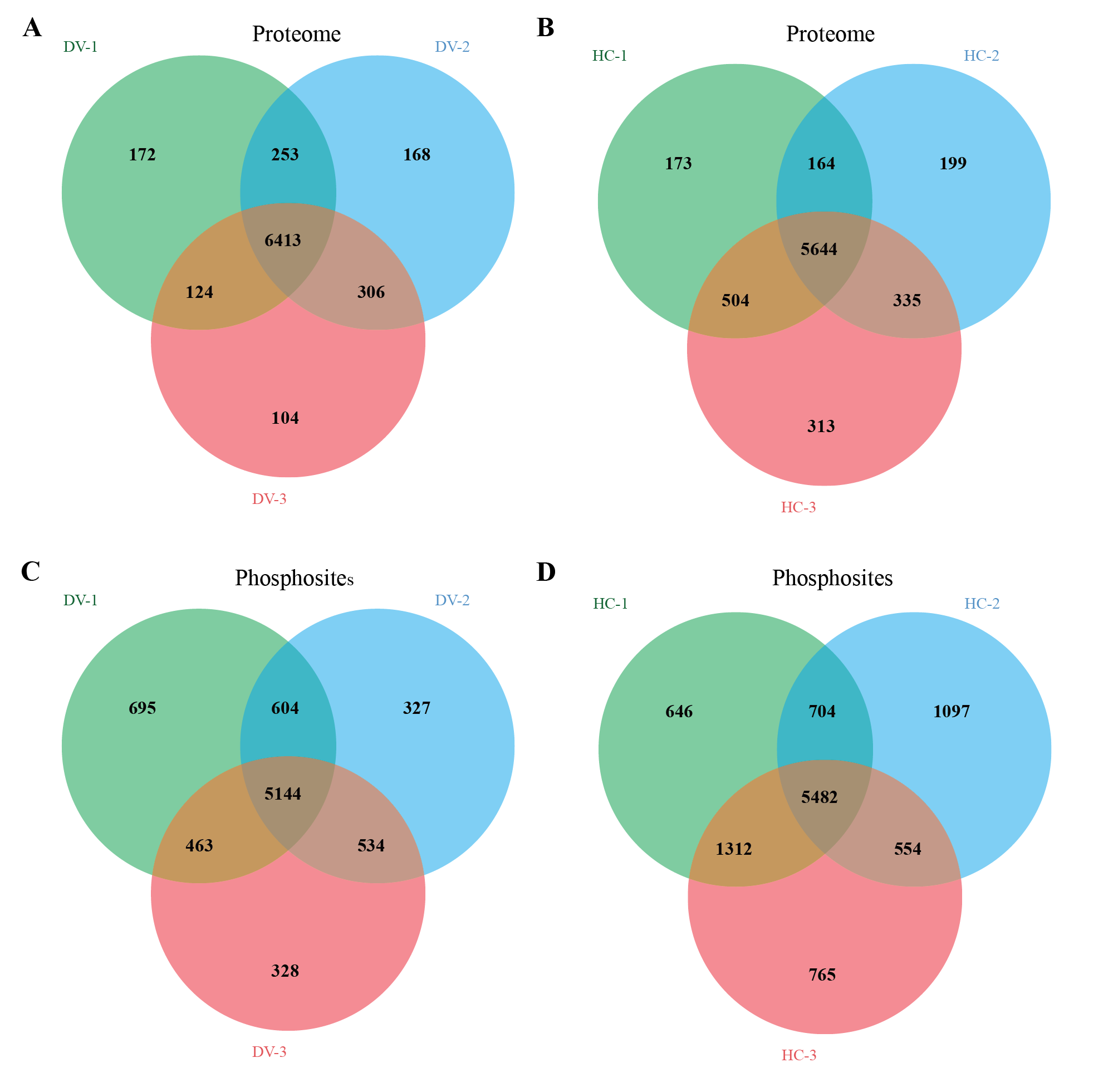


**Figure S2** Venn diagrams showing the reproducibility of proteins in liver tissues from rhesus monkeys infected with Delta strain virus **(A)** and healthy control individuals **(B)**. **(C)** and **(D)** showing the reproducibility of phosphosites in liver tissues from rhesus monkeys infected with the Delta strain virus and healthy control individuals, respectively.

**
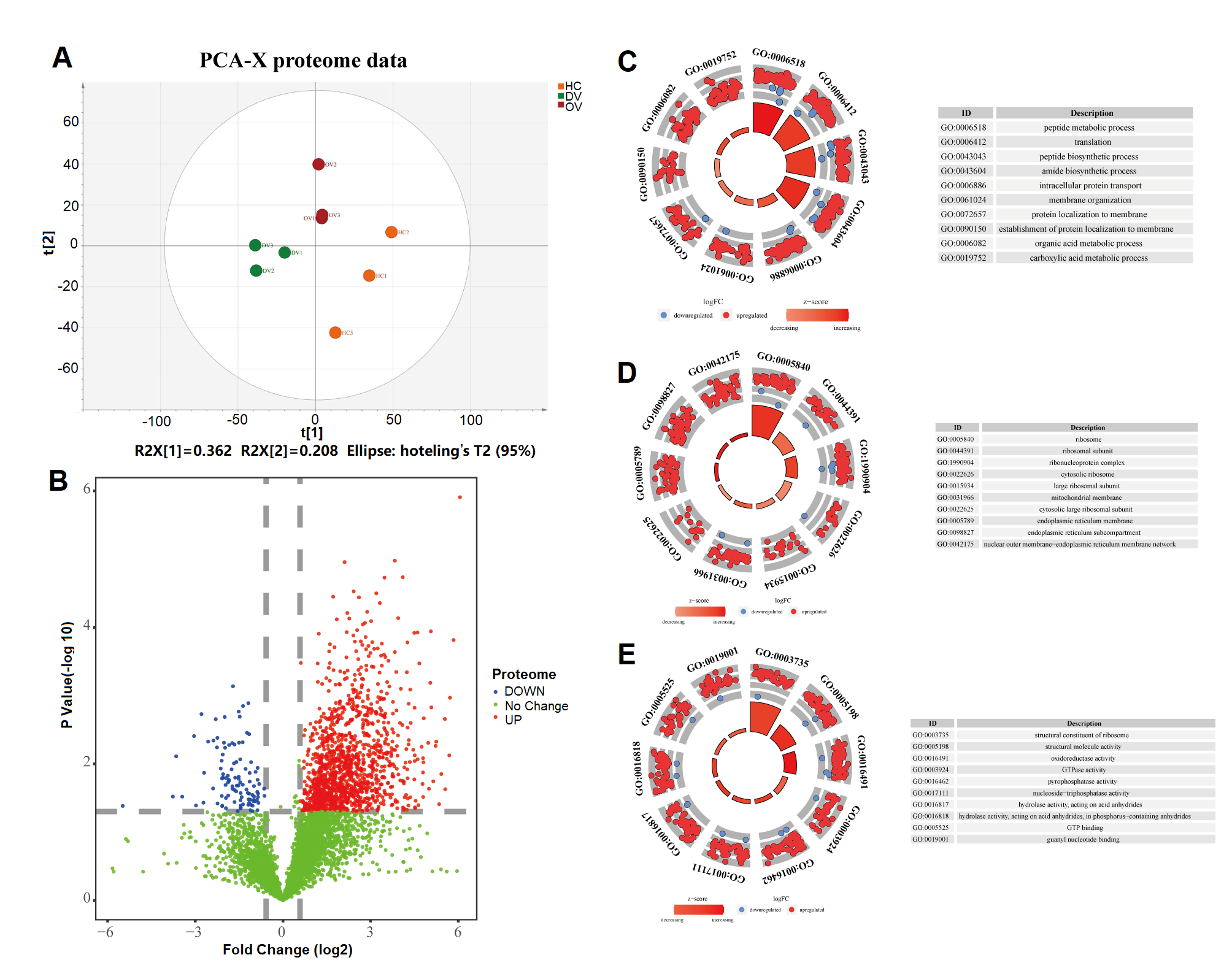
**

**Figure S3 (A)** Score plot of unsupervised PCA showing an overview of the liver proteome profile of the HC, DV, and OV groups. Each point represents one liver tissue sample. **(B)** A pairwise comparison of each protein between the HC and DV groups was performed with Student’s t-test. The p value and fold change of each quantified protein in the liver were plotted. The cutoff of differentially expressed proteins was set as a p-value < 0.05 and fold change >1.5 or <0.667. **(C-E)** Gene Ontology enrichment analysis of differently expressed proteins between the HC and DV groups. The categories of biological process (BP), cellular component (CC), and molecular function (MF) are respectively shown.


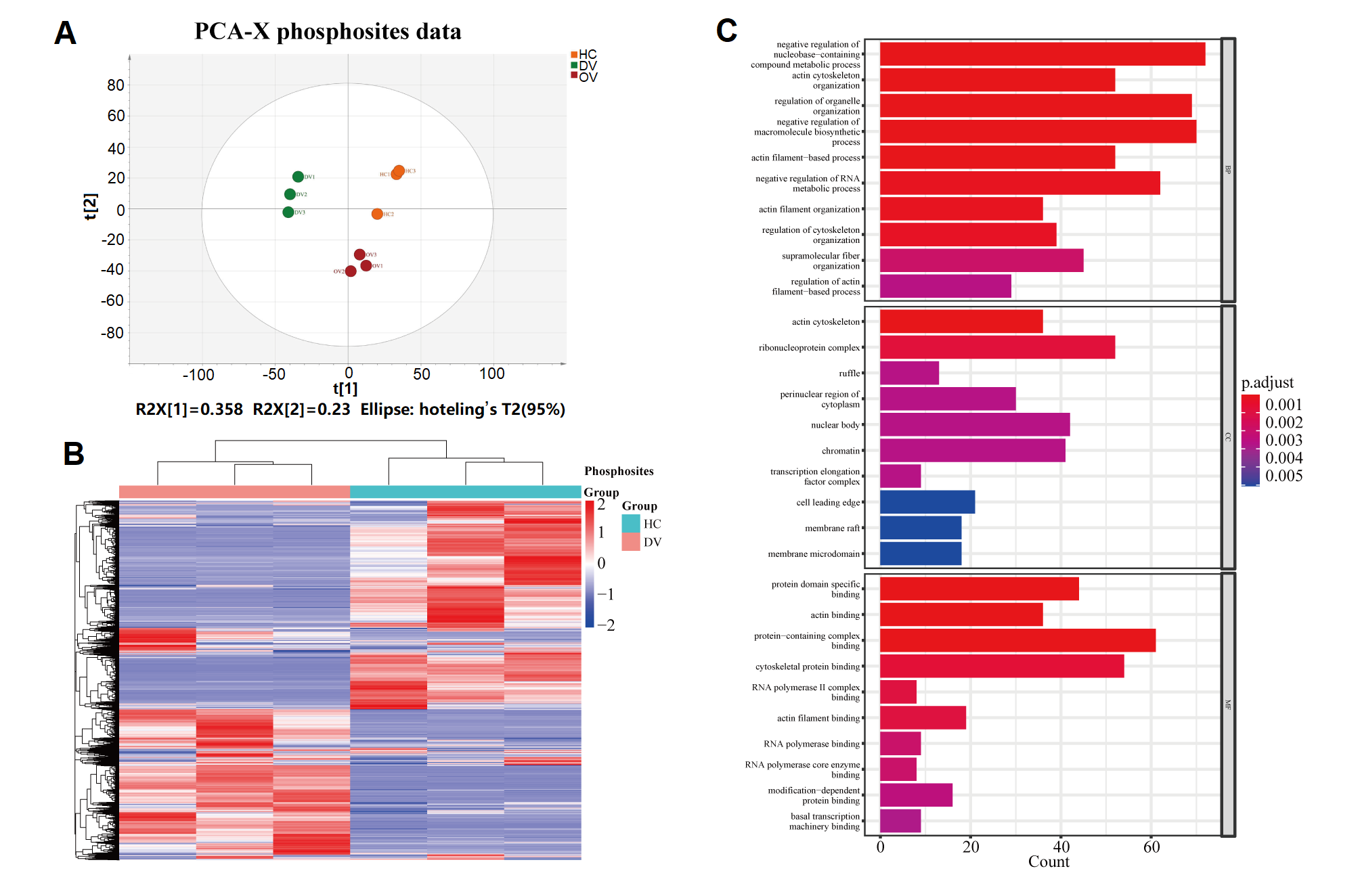


**Figure S4** Differential phosphoproteomic analysis of Delta virus-infected (DV) and original virus-infected (OV) livers from rhesus macaques. **(A)** Score plot of unsupervised PCA showing an overview of the liver phosphosites profile of the HC, DV, and OV groups. Each point represents one liver tissue sample. **(B)** The relative intensity of differently expressed phosphosites between the HC and DV group. **(C)** Gene Ontology enrichment analysis of proteins with differentially expressed phosphosites between the HC and DV groups. The categories of biological process (BP), cellular component (CC), and molecular function (MF) are respectively shown.


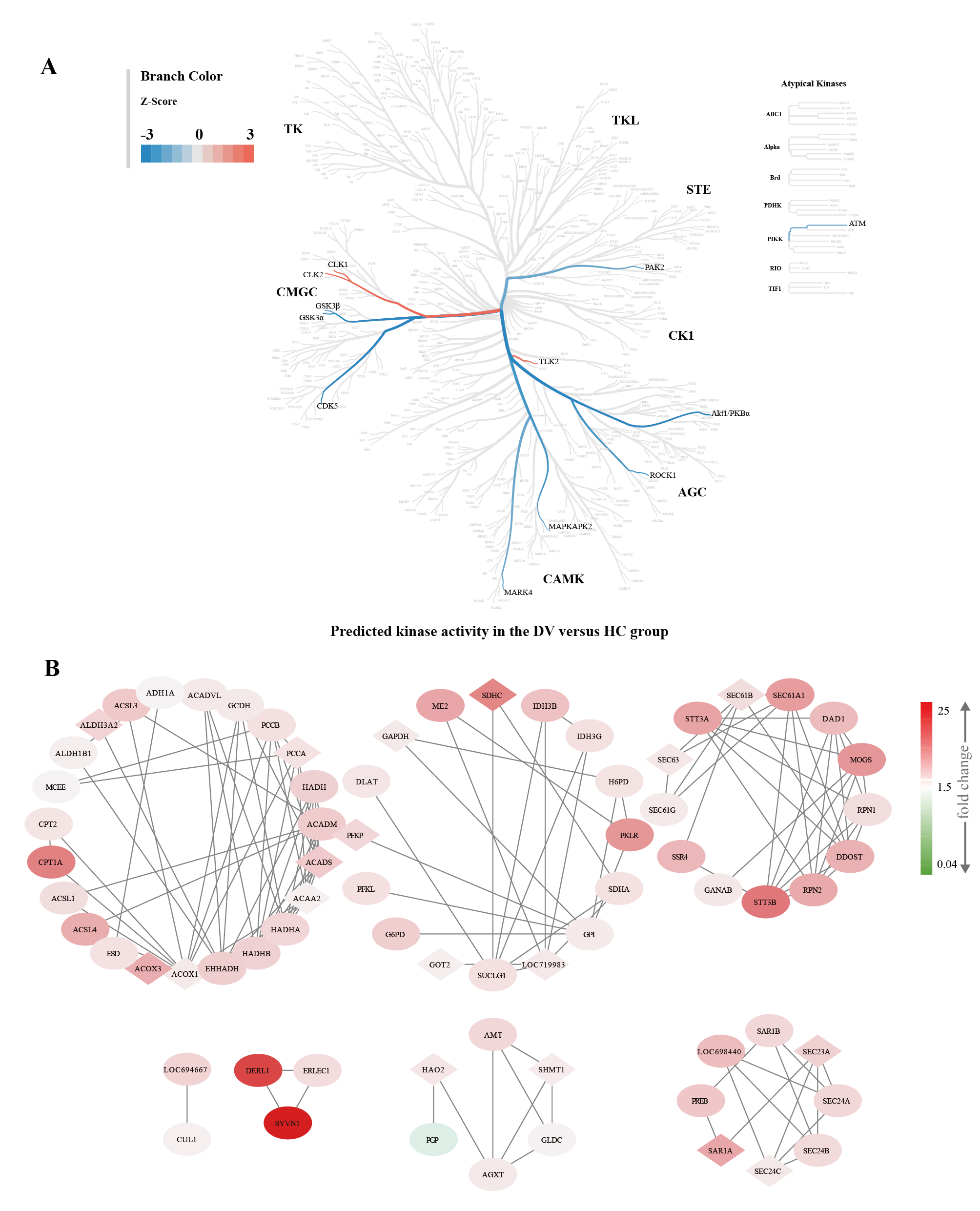


**Figure S5 (A)** Prediction of kinase activity using NetworKIN, which is based on differentially expressed phosphosites in the liver between the DV and OV groups. Blue: inhibited kinases after Delta SARS-CoV-2 infection. Orange: activated kinases after infection. **(B)** Protein-protein interaction analysis of differently expressed proteins enriched in fatty acid degradation, carbon metabolism, and protein processing in the endoplasmic reticulum. Ellipse: proteins with non-differently expressed phosphosites; Diamond: proteins with differently expressed phosphosites.


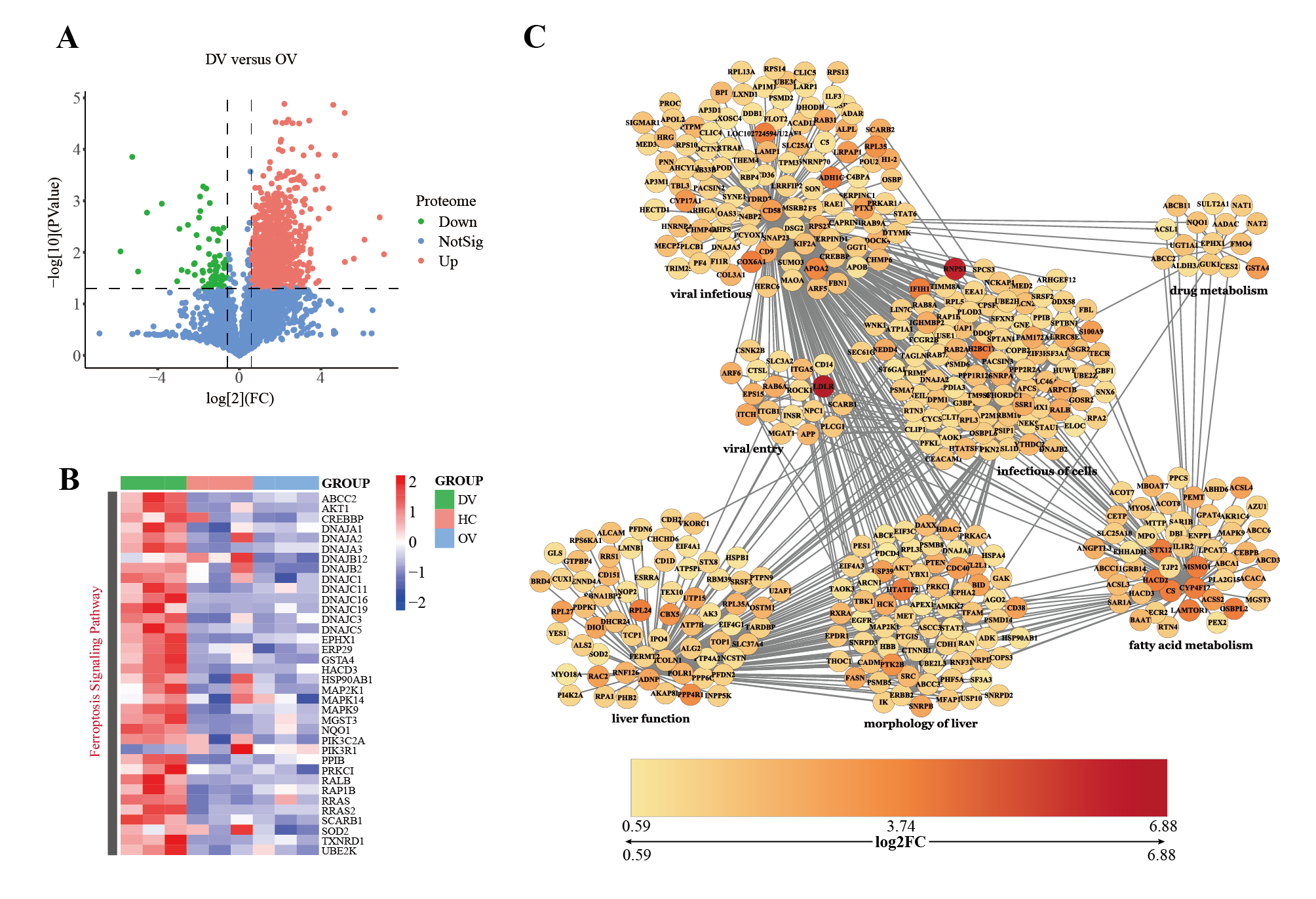


**Figure S6** Differential proteomic analysis of Delta virus-infected (DV) and original virus-infected (OV) livers from rhesus macaques. **(A)** A pairwise comparison of each protein between the DV and OV groups was performed with Student’s t-test. The p- value and fold change of each quantified protein in the liver were plotted. The cutoff of differentially expressed proteins was set as a p-value < 0.05, fold change>1.5 or <0.667. **(B)** The relative intensity of proteins enriched in the ferroptosis signaling pathway in livers in the HC, OV, and DV groups. **(C)** Interaction network of proteins that are upregulated in the livers of DV groups. Color depth indicates the high and low levels of the protein abundance ratio (log2 DV/OV).


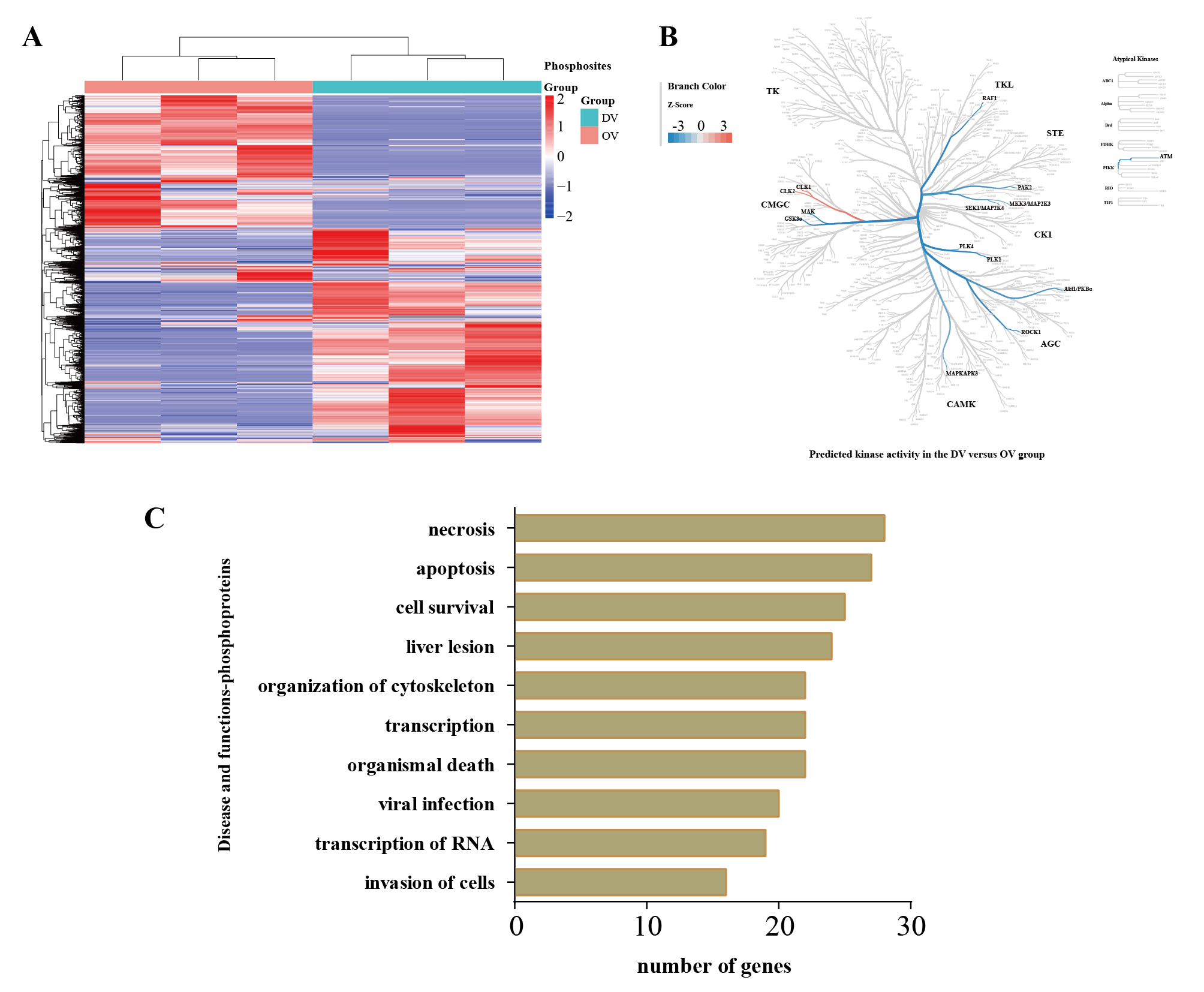


**Figure S7** Phosphoproteomic analyses of Delta virus-infected (DV) and original virus-infected (OV) livers from rhesus macaques. **(A)** The relative intensity of differently expressed phosphosites in the OV and DV groups. **(B)** Prediction of kinase activity using NetworKIN, which is based on differentially expressed phosphosites in the liver between the DV and OV groups. Blue: inhibited kinases after Delta SARS-CoV-2 infection. Orange: activated kinases after infection. **(C)** Dominant function annotation of predicted kinases in IPA.


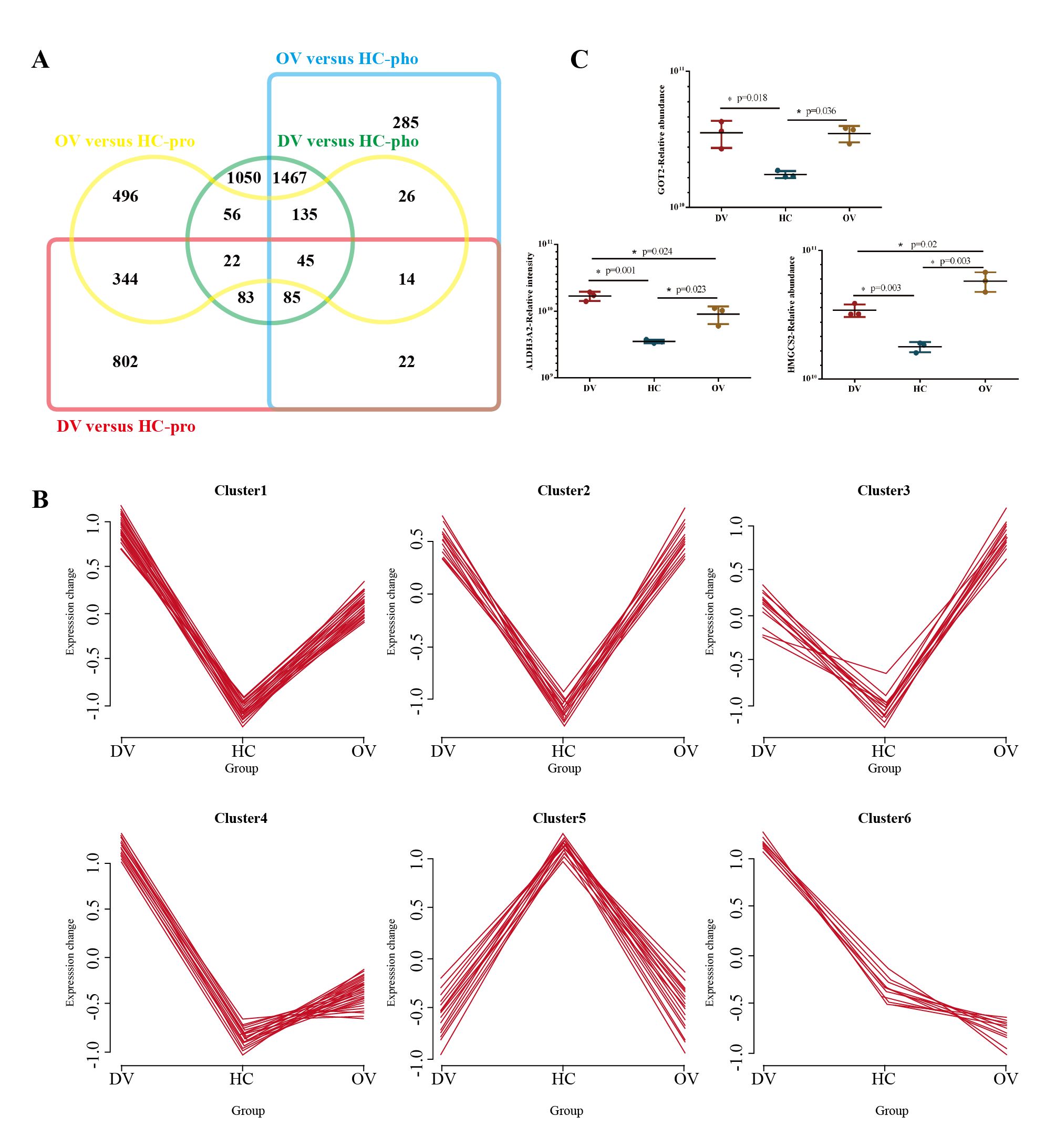


**Figure S8** Common proteomic and phosphoproteomics analysis of Delta virus-infected (DV) and original virus-infected (OV) livers of rhesus macaques. **(A)** Venn diagrams showed the overlaps of differently expressed proteins and phosphoproteins with differently expressed phosphosites when comparing two kinds of SARS-CoV-2 virus with healthy controls, respectively. **(B)** MFUZZ analysis of proteins that were differentially expressed when comparing virus-infected groups with healthy controls. **(C)** The scatter plot of the quantified values of ALDH3A2, GOT2, and HMGCS2.

**Supplementary Tables**

**Supplementary Table S1**. All quantifiable proteins in rhesus macaque livers.

**Supplementary Table S2**. Differentially expressed proteins in rhesus macaque liver samples.

**Supplementary Table S3**. Functional analysis of differentially expressed proteins in rhesus macaque livers.

**Supplementary Table S4**. All quantifiable phosphosites in rhesus macaque livers.

**Supplementary Table S5**. Differentially expressed phosphosites in rhesus macaque livers.

**Supplementary Table S6**. Predicted kinase activity in rhesus macaque livers.

**Supplementary Table S7**. Proteins for MFUZZ analysis.
